# Bridging studies in wild animal species and humans to better understand, assist, and control reproduction

**DOI:** 10.1016/j.xfre.2025.01.013

**Published:** 2025-04-15

**Authors:** Pierre Comizzoli, Richard J. Paulson

**Affiliations:** a Smithsonian’s National Zoo and Conservation Biology Institute, Washington, D.C.; b USC - HRC Fertility, Division of Reproductive Endocrinology and Infertility, Department of Obstetrics and Gynecology, Keck School of Medicine of the University of Southern California, Los Angeles, California

Reproductive processes of wild animal species stand as a perfect illustration of nature's diversity. This special issue offers to explore intriguing connections between research efforts on wild animal species and human fertility as well as the potential to drive innovative solutions to shared challenges. Although human reproductive medicine and animal reproduction are traditionally viewed as distinct fields, there is growing recognition that these domains are deeply intertwined from endocrinology to assisted reproduction (1, 2, 3). This special issue aims to catalyze dialogue and collaboration among researchers studying reproduction across species. Experts in various wild animal species were asked to reflect on lessons learned from reproduction and fertility studies in their respective fields and how it may help human reproductive medicine.

Comparative studies provide invaluable insights into the evolutionary pressures shaping reproductive strategies and highlight fundamental biological principles that go beyond species boundaries. Articles in this special issue on amphibians (authored by Vance Trudeau), birds (by George Bentley), koalas (by Steve Johnston), rhinos (by Suzannah Williams), and marine mammals (by Michelle Shero) underscore this point. Unique reproductive traits encountered in nature but not in humans, such as embryonic diapause in some mammalian species (4) or the absence of reproductive aging (5), could provide an invaluable source of inspiration to address infertility issues in humans. The article by Anneke Moresco also highlights the comparative values in the study of pathologies affecting the reproductive system.

Emerging technologies, such as gene editing, stem cell–based gametogenesis, and bioengineered reproductive tissues, hold immense promise for advancing both human and animal reproductive health. Two articles feature some of these advances: stem cell technologies (authored by Ashlee Hutchinson) and microbiomes associated with the reproductive tract and the mammary glands (by Sally Bornbusch).

Additionally, assisted reproductive technologies combined with gamete and embryo cryopreservation have revolutionized human fertility treatments. Those technologies also show promise in biodiversity conservation efforts. However, advances owe much to foundational research conducted in nonhuman species, from laboratory rodents to livestock. Conversely, wildlife conservation efforts frequently adapt assisted reproductive technology techniques developed in humans, such as gamete preservation and artificial insemination, to endangered species and their preservation (3). The reciprocal flow of knowledge between disciplines illustrates the power of collaborative and integrative research. The article by Alexandre Silva on gonadal tissue biobanking and in vitro culture is a good example of possible collaborations in that field.

Human and animal reproductive systems face diverse threats, including environmental toxins, climate change, habitat loss, and stress. These factors impact fertility rates, gestation success, and offspring survival. In wildlife populations, reproductive challenges threaten biodiversity and ecosystem stability. In humans, infertility is a rising concern, with one in six couples worldwide experiencing difficulty conceiving. Understanding the parallels between species can uncover shared vulnerabilities and pave the way for targeted interventions. The study of hormonal regulation, reproductive timing, and gamete quality across species has revealed surprising commonalities. For example, research on endocrine-disrupting chemicals has shown similar mechanisms of action in humans and wildlife, demonstrating how pollutants disrupt reproductive health universally. These findings also reinforce the need for policies that protect reproductive health at the ecosystem level. The article by Mary Ann Ottinger about the exposome is a good illustration about the need to jointly consider wildlife and human reproductive health since animal species often share the same environment and face the same issues related to changes. The last article in this Special Issue by Mayako Fujihara presents the possibility to increase collaborative efforts between human and wildlife biobanks to build a new concept of One Reproductive Health.

In some cases, controlling the reproduction of certain wild animal populations becomes necessary to maintain ecological balance and protect human interests. This is particularly true in scenarios where overpopulation leads to habitat destruction, human-wildlife conflict, or the spread of disease. Methods such as immunocontraception and sterilization are being explored as humane and effective solutions. Nonetheless, these measures raise important ethical questions about our role in nature and the long-term impacts of such interventions.

Whether safeguarding the genetic diversity of a threatened species or developing therapies for human infertility, the bridges between disciplines have the potential to transform reproductive science and its applications. We invite the reader to explore the different contributions, which highlight cutting-edge research, innovative technologies, and thought-provoking discussions. By sharing methodologies, data, and perspectives, we can accelerate progress in understanding the complexities of reproduction and addressing its challenges. Together, we can build a vision of reproductive science without boundaries, fostering a deeper understanding of life itself.

## CRediT Authorship Contribution Statement

**Pierre Comizzoli:** Conceptualization, Writing – original draft, Writing – review & editing. **Richard J. Paulson:** Conceptualization, Writing – original draft, Writing – review & editing.

## Declaration of Interests

P.C. has nothing to disclose. R.J.P. has nothing to disclose.
